# Correction: [On Becoming a Global Citizen: Transformative Learning Through Global Health Experiences]

**DOI:** 10.5334/aogh.3207

**Published:** 2021-03-16

**Authors:** Debra K. Litzelman, Adrian Gardner, Robert M. Einterz, Philip Owiti, Charity Wambui, Jordan C. Huskins, Kathleen M. Schmitt-Wendholt, Geren S. Stone, Paul O. Ayuo, Thomas S. Inui, Ann H. Cottingham, Rachel A. Umoren

**Affiliations:** 1 Indiana University (IU) School of Medicine and IU Center for Global Health, Indianapolis, IN; 2 Indiana University (IU) School of Medicine and the IU Center for Global Health, Indianapolis, IN; 3 Academic Model Providing Access to Healthcare (AMPATH) Partnership, Eldoret, Kenya; 4 Indiana University (IU) School of Medicine, Indianapolis, IN; 5 Massachusetts General Hospital Center for Global Health, Boston, Massachusetts, US; 6 Moi University College of Health Sciences, Eldoret, Kenya; 7 Indiana University (IU) Division of General Internal Medicine, Indianapolis, IN; 8 University of Washington School of Medicine, Seattle, WA

## Abstract

This article details a correction to the article: (Litzelman DK, Gardner A, Einterz RM, Owiti P, Wambui C, Huskins JC, Schmitt-Wendholt KM, Stone GS, Ayuo PO, Inui TS, Cottingham AH, Umoren RA. On Becoming a Global Citizen: Transformative Learning through Global Health Experiences. *Annals of Global Health*. 2017 May–August; 83(3–4): 596–604. DOI: *http://doi.org/10.1016/j.aogh.2017.07.005*. PMC5726429).

## Correction

Authorship Correction: (On Becoming a Global Citizen: Transformative Learning through Global Health Experiences). Ms. Ann Cottingham came forward after the paper was published requesting authorship for her contribution to the qualitative analyses and after review it was determined that her contributions were sufficient to qualify for authorship. Ms. Cottingham contributed to the qualitative analyses and reviewed and contributed to drafts of the manuscript. All authors have agreed in writing to the requested change.

Amended Author List:

Litzelman DK, Gardner A, Einterz RM, Owiti P, Wambui C, Huskins JC, Schmitt-Wendholt KM, Stone GS, Ayuo PO, Inui TS, Cottingham AH, Umoren RA. On Becoming a Global Citizen: Transformative Learning through Global Health Experiences. *Annals of Global Health*. 2017 May–August; 83(3–4): 596–604. DOI: *http://doi.org/10.1016/j.aogh.2017.07.005*. PMC5726429.

